# DNA aneuploidy as a topographic malignant transformation pattern in a pleomorphic adenoma of long-term evolution: a case report

**DOI:** 10.1186/1752-1947-5-541

**Published:** 2011-11-04

**Authors:** Lorena Gallego, Luis Junquera, Josué Hernando, Manuel F Fresno, Ana Salas, Tommaso Cutilli

**Affiliations:** 1 Department of Oral and Maxillofacial Surgery. Cabueñes Hospital, 33394, Gijón, Spain; 2 University of Oviedo, School of Dentistry, 33009, Oviedo, Spain; 3 Department of Oral and Maxillofacial Surgery, Central University Hospital, 33007, Oviedo, Spain; 4 Department of Pathology, Central University Hospital, 33007, Oviedo, Spain; 5 Cytometry Unit, Scientific-Technological Services, Central University Hospital, 33007, Oviedo, Spain; 6 Italy Health Sciences Department, Maxillofacial Surgery Unit, University of L'Aquila, 67100, L'Aquila, Italia

## Abstract

**Introduction:**

We present a case of long-term evolution of a submandibular pleomorphic adenoma. There is little information about topographic malignant transformation patterns of pleomorphic adenomas.

**Case presentation:**

We extensively analyze a giant submandibular mixed tumor of 25-year evolution in a 57-year-old Caucasian woman. Deoxyribonucleic acid ploidy was evaluated in different superficial and deep areas using flow cytometry analysis and correlated with pathological and immunohistochemical characteristics. Superficial areas exhibited a typical histological pleomorphic adenoma pattern and were deoxyribonucleic acid diploid. Deep samples showed deoxyribonucleic acid aneuploidy, atypical histological benign features and expression of markers involved at an early-stage of malignant transformation, such as tumor protein 53 and antigen Ki67.

**Conclusion:**

These findings revealed that deep tumor compartments may be involved in the initial stages of malignant transformation. Deoxyribonucleic acid ploidy analysis may provide an additional diagnosis tool and indicate 'uncertain' areas that require careful study to avoid diagnostic errors. Larger studies are needed to confirm our results and to evaluate the usefulness of the technique.

## Introduction

Pleomorphic adenomas (PAs) are the most common benign tumors arising in salivary glands and their malignant transformation to carcinoma ex pleomorphic adenomas (CXPAs) accounts for between 4.5% and 15% of all cancers of these glands [1]. The diagnosis of CXPA is based on the coexistence of epithelial malignancy with histologically benign PA [2]. These tumors are typically considered as high-grade carcinomas, with frequent metastases and disease-related deaths [3].

Long-term evolution of a PA might increase the risk of malignant transformation [4]. Although rare, cases of giant PA have been reported, most of them involving the parotid gland [5-7]. Misdiagnosis is not rare in these cases, because the CXPA component may be small and therefore missed on histological analysis. There are few series published in the literature focusing on ploidy analysis for the prediction of tumor aggressiveness and for differentiating benign from malignant salivary gland tumors [8-10]. However, a lack of correlation between cytometric parameters and histological or immunohistochemical parameters has not been described.

The objective of this case report was to analyze a giant PA tumor of 25-year evolution. Deoxyribonucleic acid (DNA) ploidy was evaluated in different areas using flow cytometry analysis, and correlated with pathological and immunohistochemical characteristics in order to assess whether ploidy analysis may improve the diagnostic accuracy for predicting malignancy.

## Case presentation

**Figure 1:**
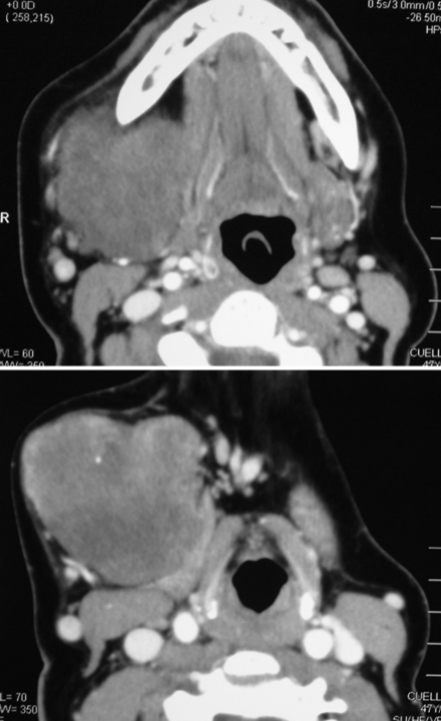
**Preoperative computed tomography scan demonstrating a large well-defined mass without cystic changes or necrotic areas in the right submandibular region**.

A 57-year-old Caucasian woman presented with a large non-tender submandibular mass. The lesion had been present for about 25 years with a slow increase in size. Our patient's medical history was unremarkable. A physical examination revealed a giant painless, movable, semi-hard elastic mass in her right submandibular region measuring about 8×6 cm. Fine needle aspiration cytology was suggestive of a PA. Contrast-enhancement computerized tomography confirmed a giant well-defined mass without cystic changes in her right submandibular region (Figure 1). No lymph node swelling or other tumorous lesions were detected. Submandibular tumor extirpation was performed under general anesthesia.

**Figure 2:**
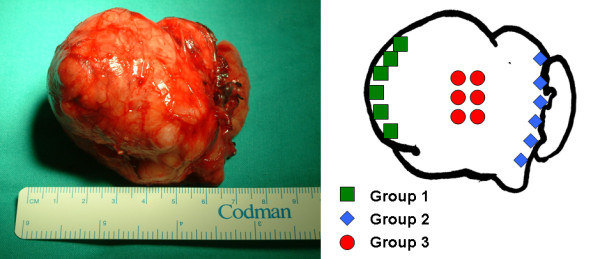
**Macroscopic overview of the resected pleomorphic adenoma and topographic mapping of the samples obtained**. Group 1: superficial samples adjacent to subcutaneous tissue; group 2: superficial samples adjacent to the floor of the mouth; group 3: deep samples.

Immediately after removal, samples were obtained in the surgery room following a topographic scheme (Figure 2). Group 1 comprised six samples from the periphery of the tumor, adjacent to the subcutaneous tissue; group 2 comprised six samples from the periphery of the tumor, adjacent to the floor of the mouth; and for group 3, the tumor was opened along the midline, and six deep samples were extracted from the center of the tumor.

Half of the samples in each group were fixed in 4% buffered formalin, processed and embedded in paraffin according to routine procedures, for histological and immunohistochemical analysis. The remainder of the samples of fresh material for each group were immediately submitted for DNA flow cytometry. The rest of the surgical specimen was routinely studied in the Department of Pathology and diagnosed as a benign PA.

Samples of each group were minced with a scalpel in phosphate-buffered saline solution. Single nuclear suspensions were prepared by filtering through a 50-μm nylon mesh. The DNA contents were measured in a Cytomics FC500 (Beckman Coulter Inc., Fullerton, CA, USA) flow cytometer. DNA histograms of at least 10, 000 nuclei were plotted. The DNA-diploid cell population corresponding to surrounding normal tissue from the same location was used as an internal reference standard for the identification of DNA-aneuploid clones. The percentages of the cell cycle phases as well as the DNA indices of the aneuploid clones were calculated using the Modfit 5.2 software package. DNA histograms were classified as diploid if there was a single G0-G1 peak and aneuploid if additional G0-G1 peaks were present. The ratio of aneuploid G0-G1 peak values to diploid G0-G1 peak values was expressed as a DNA index. All specimens had a G0-G1 peak coefficient of variation of no more than 4%. The following were taken as cytometric variables: DNA ploidy, DNA index, and S-phase fraction. The cases with DNA indices between 0.9 and 1.10 were considered as DNA diploids, and those less than 0.9 or greater than 1.10 as DNA aneuploids.

Half of the paraffin-embedded samples of all three groups were routinely stained with hematoxylin and eosin. The rest of the paraffin samples were submitted to the labeled-polymer method of immunohistochemistry using antibodies against α-smooth-muscle actin (α-SMA), cytokeratin (CK) AE1/AE3, CK 8, protein 53 (p53), protein 63 (p63) and antigen Ki67.

**Figure 3:**
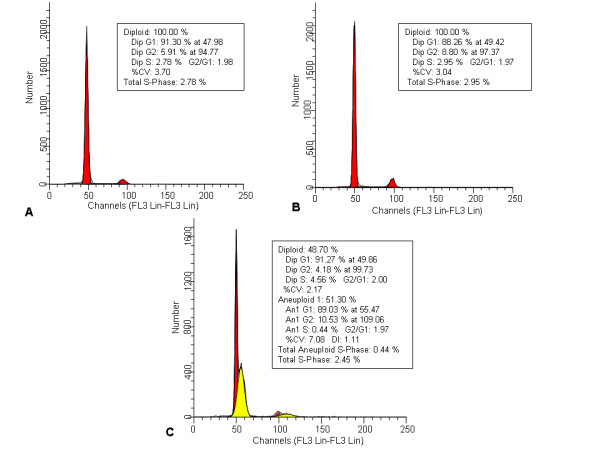
**Histograms from flow cytometry on the fresh material**. **(A) **Histogram of group 1 samples showing a single peak in the G0-G1 area (DNA diploid). **(B) **Histogram of group 2 samples also exhibiting a diploid pattern. **(C) **Histogram of group 3 (deep) samples demonstrating DNA aneuploidy.

The histogram of Group 1 samples presented a single peak in the G0-G1 area. The cell nuclei population was 5.91% in the G2 region and 91.30% in the G1 area. The proportion of cells in the S-phase was 2.78% and the coefficient of variance (CV) was 3.70%. Samples of this group were considered as being DNA diploid (Figure 3A). Group 2 samples also exhibited a DNA diploid pattern with an 88.26% nuclei population in the G1 region and 8.80% in the G2 area. The S-phase fraction was 2.95% and the CV was 3.04% (Figure 3B). Group 3 samples showed DNA aneuploidy: 48.70% of the cell population was considered diploid with 4.18% in the G2 region, 91.27% in the G1 area and a CV of 2.17%, whereas 51.30% of the cells analyzed presented an aneuploid pattern with 10.53% in the G2 region, 89.03% in the G1 area and a CV of 7.08%. The total aneuploid S-phase was 0.44% and the total S-phase fraction was 2.45% (Figure 3C).

**Figure 4:**
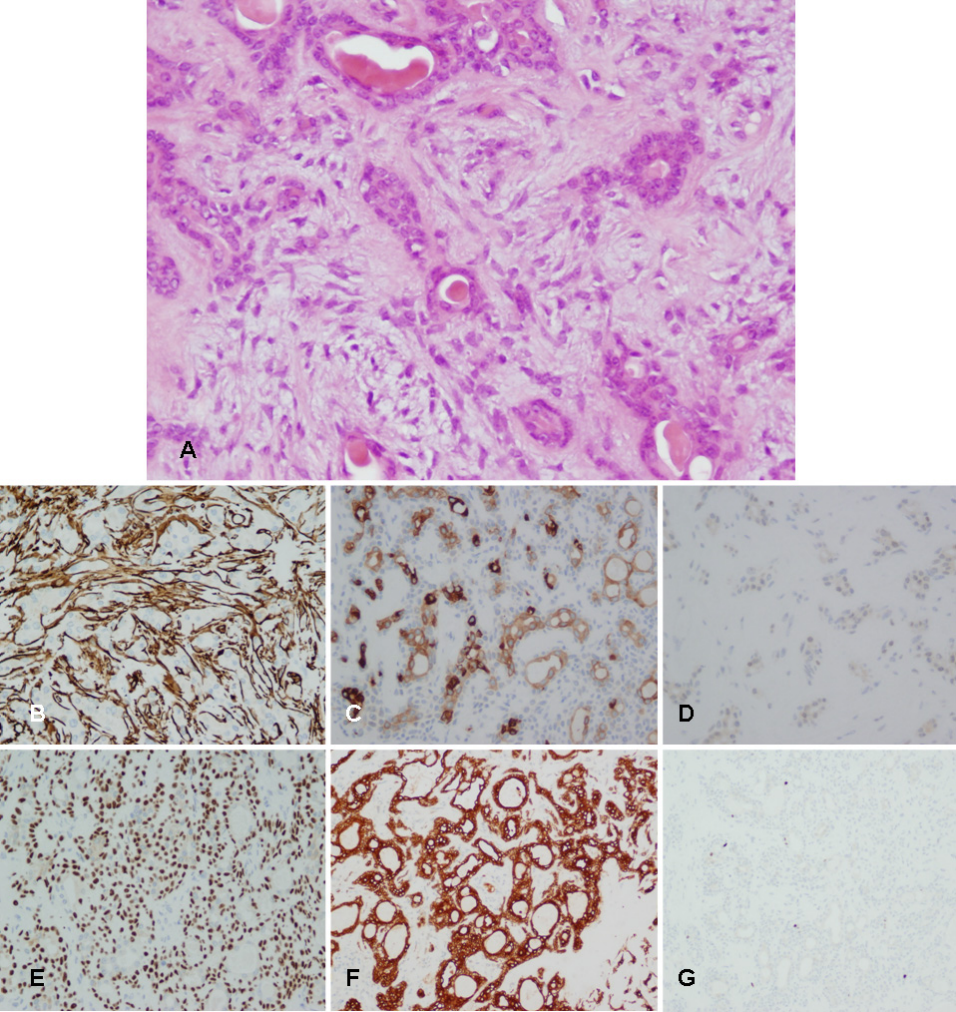
**Histological and immunohistochemical evaluation of superficial areas (groups 1 and 2)**. **(A) **Hematoxylin and eosin staining showing cords and islands of polygonal cells without atypia in a myxoid stroma (200× magnification). **(B) **Basal and myoepithelial cells highly exhibited α-SMA (200× magnification). **(C) **Slight positivity with CK 8 was observed (200× magnification). **(D) **No expression was observed with p53 (200× magnification). **(E) **Basal and myoepithelial cells highly exhibited p63 (200× magnification). **(F) **Slight positivity with CKs AE1/AE3 was observed (200× magnification). **(G) **Only occasional cells were positive with antigen Ki67 (100× magnification).

Histological analysis of Group 1 and Group 2 samples showed ductal structures, cords and islands of polygonal cells without atypia, sheets and strands of hyaline or plasmacytoid cells in a myxoid stroma. These findings were consistent with PA (Figure 4A). On immunohistochemistry, slight positivity was observed in the ductal cells with CK AE1/AE3 and CK 8 (Figure 4C-F). The non-luminal cells strongly expressed α-SMA (Figure 4B). Occasional cells were positive with proliferation antigen Ki67 and no expression was observed with p53 (Figure 4D-G). Otherwise, myoepithelial cells showed high positive nuclear staining for p63 (Figure 4E).

**Figure 5:**
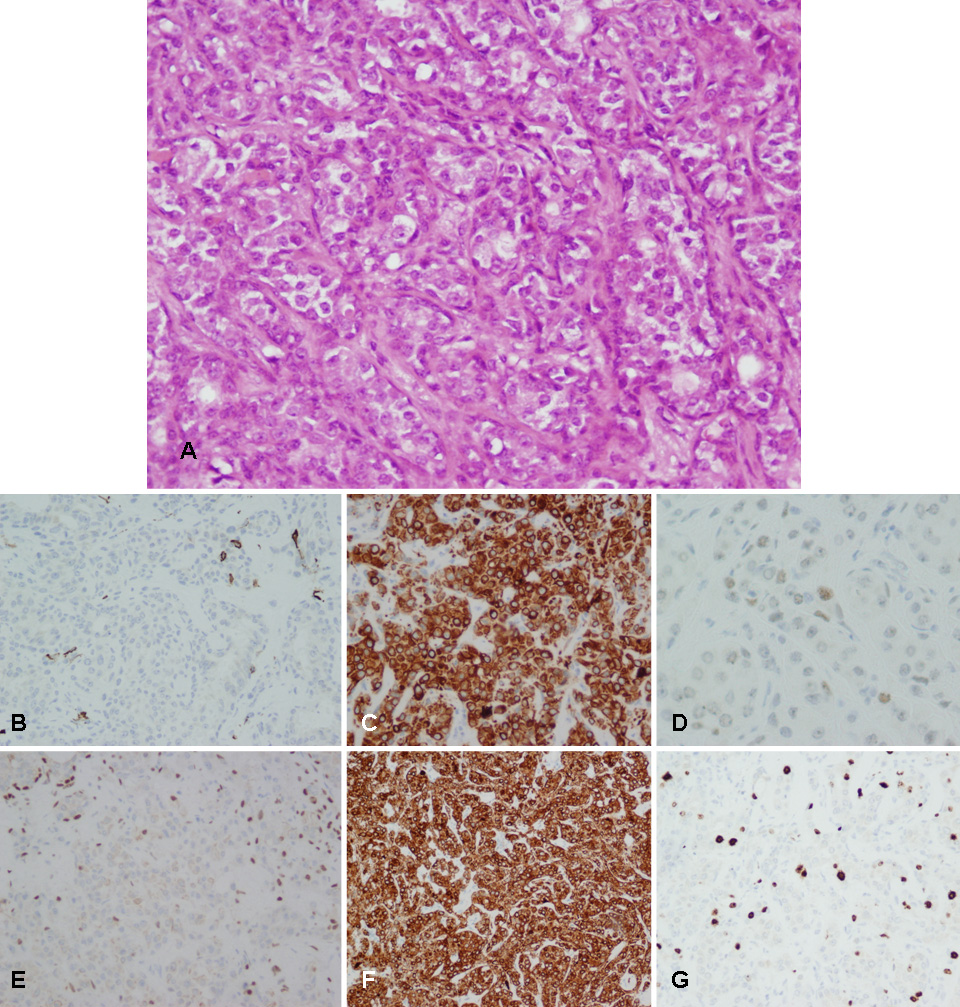
**Histological and immunohistochemical evaluation of the deep area (group 3)**. **(A) **Hematoxylin and eosin staining showing hypercellular areas composed of round to ovoid epithelial cells, without nuclear pleomorphism or atypia (200× magnification). **(B) **Immunostaining with α-SMA was only detected in a few myoepithelial cells (100× magnification). **(C) **CK 8 was strongly expressed in most of the tumoral cells (200× magnification). **(D) **Positivity to p53 was detected in a few epithelial cells (200× magnification). **(E) **Immunostaining with p63 was only detected in a few myoepithelial cells (100× magnification). **(F) **CKs AE1/AE3 were strongly expressed in most of the tumoral cells (100× magnification). **(G) **Stronger expression of Ki67 antigen than in groups 1 and 2 was observed (100× magnification).

Group 3 samples exhibited an unusual histological pattern. These hypercellular areas were composed of blocks of round to ovoid epithelial cells without the 'reminiscent' myoepithelium. The epithelial cells were round with pale eosinophilic cytoplasm and round to oval nuclei. Nuclear pleomorphism or atypia, malignant luminal cells and necrotic foci were not observed (Figure 5A). The immunohistochemical study showed strong expression of CKs AE1/AE3 and CK 8 in most of the epithelial cells (Figure 5C-F). p63 and α-SMA staining were seen to a lesser degree than in the Group 1 and 2 samples (Figure 5B-E) whereas expression of antigen Ki67 was more intense (Figure 5G). p53 was expressed in a few epithelial cells (Figure 5D).

## Discussion

PA is the most common benign salivary gland tumor [1,2]. It is derived from the epithelium and typically presents as a cytologically benign circumscribed mass with variable encapsulation. This tumor is one of the few benign neoplasms than can undergo malignant transformation. The propensity for malignant transformation has been documented in the literature at 1.9% to 23.3% and CXPA represents approximately 12% of malignant neoplasms [2,11,12]. Otherwise, it has been estimated that approximately 25% of untreated mixed tumors would eventually develop into carcinomas [13]. The likelihood of a malignant change in a PA increases with the duration of the tumor and with the age of the patient. The diagnosis of CXPA requires the presence of either a recognizable mixed tumor in association with a carcinoma or carcinoma developing as a recurrent neoplasm at the site of a previous mixed tumor. Criteria for malignancy include invasiveness, cellular anaplasia or pleomorphism, atypical mitosis and abnormal architectural patterns [1,3].

It is currently postulated that malignant transformation is accompanied by genomic instability (cytogenetic and/or cytometric aneuploidy) [14]. DNA ploidy analysis has proven to be a useful prognostic indicator in a variety of salivary gland neoplasms. Abnormal DNA content has been related to aggressive behavior in adenoid cystic carcinomas, acinic cell carcinomas, mucoepidermoid carcinomas and oncocytomas [8,9]. Otherwise, most likely due to the low incidence of malignant salivary gland tumors, only a few attempts have been made to employ DNA ploidy analysis for diagnostic assessment [10,15]. One purpose of the present study was to determine whether DNA ploidy analysis is a good diagnostic tool to distinguish malignant areas in PAs even before histopathological correlation.

Some authors reported that DNA diploidy may be seen in both benign and malignant lesions, but aneuploidy is mainly seen in malignant lesions. In the majority of previous studies, the PAs revealed a diploid pattern [8,9,15]. However, Martin *et al*. examined a series of 16 mixed tumors and found DNA aneuploidy in four cases, three of which were recurrent lesions [16]. In our tumor, deep samples (Group 3) demonstrated aneuploidy correlated with atypical histological and immunohistochemical features. This central area exhibited a hypercellular pattern, with a predominantly epithelial pattern and a lack or poor myoepithelial cells and stroma. Immunohistochemistry for CKs AE1/AE3 and CK 8 confirmed the glandular (luminal) differentiation of the epithelial portion of this tumor. Otherwise, p63 antibody and α-SMA have been shown to be reliable myoepithelial and basal cell markers, and presented minor staining in the deep area. PA typical areas, the Group 1 and 2 samples, characteristically show a variable amount of myxochondroid stroma produced by myoepithelial cells. Chau and Radden studied 53 cases of intraoral PA, and reported that stroma-poor PAs were larger than stroma-rich ones, and suggested that cellular tumors may grow at a faster rate [17]. Other studies described that, although the myoepithelial component predominates in most PAs, the genetic changes leading to malignant transformation occur more frequently in ductal luminal cells than in myoepithelial cells [18,19].

In addition, p53 and Ki67 presented higher expression in deep compared to superficial areas. It has been suggested that the index of p53 and Ki67 accumulation could be a useful biomarker for detecting tumors at an early phase of malignant transformation and distinguish PA from CXPA areas [18]. Therefore, Group 3 samples presented a hypercellular benign pattern, without dysplasia or obvious malignant change, but also showed DNA aneuploidy and expressed markers involved at an early stage of malignant transformation. It is tempting to postulate a future progression to CXPA in this area.

## Conclusion

There is little information in the literature regarding topographic malignant transformation patterns of PA. This study revealed that deep tumor compartments may be involved in the initial stages of malignant transformation. The authors of the present study suggest that deep areas in long-term evolution PAs should be carefully assessed by serial sectioning to document evidence of malignancy. In conclusion, DNA ploidy analysis may provide an additional diagnosis tool for 'uncertain' areas. The prognosis and future therapy will depend on careful study of these lesions. Obviously, more cases are needed to evaluate these results and better understand this entity.

## Abbreviations

α-SMA: smooth-muscle actin; CK: cytokeratin; CV: coefficient of variance; CXPA: carcinoma ex pleomorphic adenoma; PA: pleomorphic adenoma.

## Consent

Written informed consent was obtained from the patient for publication of this case report and any accompanying images. A copy of the written consent is available for review by the Editor-in-Chief of this journal.

## Competing interests

The authors declare that they have no competing interests.

## Authors' contributions

LG was a major contributor in writing the manuscript. LJ was the main surgeon, reviewed the patient's notes, collected the data and corrected the manuscript. MFF performed histological and immunohistochemical diagnosis and photographs. AS performed and evaluated the cytometry analysis. TC reviewed and corrected the manuscript and grammar. All authors read and approved the final manuscript.
